# Cortical Response Variation with Social and Non-Social Affective Touch Processing in the Glabrous and Hairy Skin of the Leg: A Pilot fMRI Study

**DOI:** 10.3390/s23187881

**Published:** 2023-09-14

**Authors:** Larisa Mayorova, Galina Portnova, Ivan Skorokhodov

**Affiliations:** 1 Laboratory of Physiology of Sensory Systems, Institute of Higher Nervous Activity and Neurophysiology of Russian Academy of Science, 117485 Moscow, Russia; 2 Laboratory for the Study of Tactile Communication, Pushkin State Russian Language Institute, 117485 Moscow, Russia; 3 Laboratory of Human Higher Nervous Activity, Institute of Higher Nervous Activity and Neurophysiology of Russian Academy of Science, 117485 Moscow, Russia

**Keywords:** C-tactile fibers, affective touch, afferent, glabrous skin, hairy skin, fMRI, leg (lower limb)

## Abstract

Despite the crucial role of touch in social development and its importance for social interactions, there has been very little functional magnetic resonance imaging (fMRI) research on brain mechanisms underlying social touch processing. Moreover, there has been very little research on the perception of social touch in the lower extremities in humans, even though this information could expand our understanding of the mechanisms of the c-tactile system. Here, variations in the neural response to stimulation by social and non-social affective leg touch were investigated using fMRI. Participants were subjected to slow a (at 3–5 cm/s) stroking social touch (hand, skin-to-skin) and a non-social touch (peacock feather) to the hairy skin of the shin and to the glabrous skin of the foot sole. Stimulation of the glabrous skin of the foot sole, regardless of the type of stimulus, elicited a much more widespread cortical response, including structures such as the medial segment of precentral gyri, left precentral gyrus, bilateral putamen, anterior insula, left postcentral gyrus, right thalamus, and pallidum. Stimulation of the hairy skin of the shin elicited a relatively greater response in the left middle cingulate gyrus, left angular gyrus, left frontal eye field, bilateral anterior prefrontal cortex, and left frontal pole. Activation of brain structures, some of which belong to the “social brain”—the pre- and postcentral gyri bilaterally, superior and middle occipital gyri bilaterally, left middle and superior temporal gyri, right anterior cingulate gyrus and caudate, left middle and inferior frontal gyri, and left lateral ventricle area, was associated with the perception of non-social stimuli in the leg. The left medial segment of pre- and postcentral gyri, left postcentral gyrus and precuneus, bilateral parietal operculum, right planum temporale, left central operculum, and left thalamus proper showed greater activation for social tactile touch. There are regions in the cerebral cortex that responded specifically to hand and feather touch in the foot sole region. These areas included the posterior insula, precentral gyrus; putamen, pallidum and anterior insula; superior parietal cortex; transverse temporal gyrus and parietal operculum, supramarginal gyrus and planum temporale. Subjective assessment of stimulus ticklishness was related to activation of the left cuneal region. Our results make some contribution to understanding the physiology of the perception of social and non-social tactile stimuli and the CT system, including its evolution, and they have clinical impact in terms of environmental enrichment.

## 1. Introduction

Over the past 25 years, research in sensory neurophysiology has proven the existence of a specialized system of tactile perception that integrates highly sensitive mechanoreceptors associated with non-myelinated type C fibers (CT afferents) [1]. The most intensive response of this system is observed to light, slow touches associated with positive emotions and feelings of comfort, calmness, safety, tenderness, love, and care [2]. As studies on humans and mammals have shown, the CT system plays an important role in the formation of the emotional sphere and social relations [3,4].

Fundamental physiological and clinical studies performed using neurobiological and psychophysical methods have made it possible to determine the main structural and functional characteristics of the CT afferents system in humans [1]. However, the central mechanisms of implementation of the CT system response still need to be studied.

It is believed that touch is realized by nerve impulse conduction in two ways: by fast Aβ-afferents (carrying information about physical properties of a tactile stimulus) and by slow C-tactile afferents (activated most of all by gentle, slow, caress-like stroking by tactile stimuli at temperatures near those of human skin) [1,4,5,6]. It is believed that CT afferents are found only in hairy skin but not the glabrous skin of the palm [7]. Accordingly, experimental stimulation generally has three components: distinguishing the physical properties of the stimulus and the emotional and social/interpersonal/communicative (in the case of stroking by a person’s hand) components of the stimulus.

According to available neuroimaging data (fMRI), during tactile stimulation, which has a socio-affective component (CT-optimal touch), brain structures such as the primary (SI) and secondary somatosensory cortex (SII) [8,9], insular cortex [10], posterior superior temporal sulcus (pSTS) [3,8,9,11], medial prefrontal cortex (mPFC), orbitofrontal cortex (OFC) and amygdala [9], pregenual anterior cingulate cortex (pgACC) [3,12], and dorsolateral prefrontal cortex (DLPFC) [11] are activated. Furthermore, there seems to be a stable pattern of maturation of cortical CT-sensitivity areas [8]. Different reactions in the postcentral gyrus, the middle temporal gyrus, and the insula to affective and non-affective touching are detected as early as 11–36 days [13] and 2 months of age, respectively [14].

Returning to the components of CT stimulation, the above-mentioned brain areas appear to respond selectively to physical, affective, and social/communicative stimulus parameters. Thus, touch discrimination, according to the literature, is realized in the primary and secondary somatosensory cortex, where most of the Aβ afferents arrive [15,16,17,18,19]. Where the affective component of touch is processed is currently unknown. Existing sparse evidence has emphasized the role of the anterior cingulate cortex [15] and insula [20] in this process. However, these data need to be clarified, including which other areas may be involved in the processing of the affective aspect of touch.

However, in most neuroimaging studies, except the studies of Lindgren et al. (2012), Boehme R. et al. (2019), Strauss et al. (2019), and Kress et al. (2011) [12,21,22,23], exposure was conducted using a soft brush, which eliminated the interpersonal effect and thus reduced the possibility of investigating the social component directly. In addition, these studies generally used control stimulation, which does not allow for the separation of the affective and social components.

Additionally, in all the above-mentioned studies of the CT system, except for Tuulari et al. (2019) [13] (stimulation of leg), stimulation was performed in the arm area. Social tactile contacts in humans are realized in most cases by touching the hand and shoulder area, and the vast majority of works have been carried out with stimulation of these areas. CT-afferents have been found on the face [24] and in hairy skin such as in the fore- arm, in humans [1,2,25,26].

Little is known about the central mechanisms of the CT system functioning in other parts of the body. At the same time, these data could contribute to understanding of the origin and development of the system of social tactile perception in the human brain. By studying C-tactile representations from skin that has not become a “social tactile organ” (in humans, the lower extremities), we can observe the C-tactile system in the state it was in before its inclusion in the process of interpersonal interaction (assuming that the physiological basis was evenly distributed throughout the body). Regarding this connection, the aim of our study was to reveal which brain regions specifically respond to the social component activated by perception of slow gentle strokes with the hand in the lower limb area. We used stimulation of the hairy part of the shin, as well as stimulation of the foot sole. In selecting the type of stimulus, we proceeded from the assumption that both stimuli should evoke an emotional response but would differ strikingly in their social component. Thus, we settled on two types of strokes: the socially affective touch (gentle slow stroking of the hand without glove) and the affective but non-social touch (slow stroking with a peacock feather).

Since the CT system was only recently discovered [27], the research on the mechanisms that regulate the transmission of information along CT afferent fibers is ongoing. It should be noted, however, that different parts of the human body have been studied to varying degrees in terms of their C-tactile afferentation. In particular, the regulation of the CT system’s response to pleasant social stroking by the hand had received the most attention [28,29]. In contrast, much less research has been performed on the mechanisms of information transmission about C-tactile stimulation from the back, abdomen, and legs. However, touching the hand differs in degree of intimacy from touching the foot or belly according to their social and personal significance [30].

When preparing research based on prior data describing the different concentrations of C-tactile afferents in the hairy and non-hairy parts of the body, we, on the one hand, hypothesized that social touches would induce different responses in the sole and shin, and we aimed to describe them. On the other hand, we presumptively decided to investigate the central mechanisms of the CT system’s response to touch for a region of the body that was essentially unstudied in terms of neuroimaging, so we started with potential distinctions between the innervation of the arms and legs. The third independent objective of our research was to investigate the tickling sensations and their neural basis during stimulation by a peacock feather of the feet soles and shins.

## 2. Methods

### 2.1. Study Participants

Twenty-six healthy volunteers (12 men, 14 women) between 18 and 40 years of age participated in the study. The inclusion criteria: age from 20 to 40 years old, male or female, right-handed, no history of neurological or psychiatric diseases, no drugs before the study, written informed consent, native Russian speakers, no alcohol for at least 48 h before the study, and no smoking or caffeine use for at least 2 h before study The exclusion criteria: the use of neuroleptics (antipsychotics), anxiolytics, mood stabilizers, antidepressants, psychostimulants, or nootropics; history of claustrophobia; implanted metal clips or wires; right leg fractures; leg epilation, waxing, or shaving within 24 h before the examination; influence; and Touch Experiences and Attitudes Questionnaire (TEAQ-37) score <100. All subjects signed an informed consent form to participate in the study. Before scanning, the subjects were warned about the nature of the stimulation being presented. Two to three days before the study, we informed participants that they should not epilate, wax, or shave the legs, and we explained some details of the experimental procedure: (1) we will present tactile strokes during fMRI recording; and (2) you will be asked to complete the TEAQ-37 to exclude touch perception abnormalities [31]. The subjects were required to carefully read and sign an informed consent form before the study, empty their pockets of all metal objects, and turn off any electronic devices.

### 2.2. Experimental Design and Stimuli

The passive fMRI task included four types of strokes: of the shin with a feather, of the shin with the hand, of the foot sole with the feather, and of the foot sole with the hand. We used a peacock feather. Stimuli were presented in blocks of 9 s in random order. Using a pseudo-random number generator, we created pseudo-random sequences, in which each of the four types of stimulation was assigned a number: 1—feather stimulation of the shin; 2—feather stimulation of the sole of the foot; 3—hand stimulation of the sole of the foot; and 4—hand stimulation of the shin. Each participant in the study was presented with his or her own randomized sequence. A 6-s rest was provided between blocks. A total of 12 blocks of each stimulus type were presented. The total duration of stimulation was 12 min. Manual stroking was performed by a specially trained operator under the supervision of video instructions. The same clinical psychologist, who is female, conducted the study. The experimenter underwent specialized training, in which he learned how to present stimulation with the same speed and pressing force. The rate of stroking for all types and regions of stimulation was the same and was 3–5 cm/s, which has been rated by adult human subjects as the most pleasant (CT-optimal). The size of touched area was 20 cm, and skin-to-skin touching was performed with the full flat hand. The operator accurately followed the video instructions to achieve speed uniformity in the presentation of the stimuli. The right shin and sole were stroked.

Since there was direct bodily contact between two people in our research paradigm, we hypothesized that hand stimulation of the shin (and to a lesser extent, the foot sole) was the most socially significant stimulation.

We did not force subjects to close or keep their eyes open intentionally to avoid systematic influence of this factor. When subjects asked about it, we suggested that they choose the mode that was most comfortable for them.

### 2.3. Functional MRI Scanning Parameters

Functional and anatomical images were acquired using a 3.0-T Philips Achieva (Koninklijke Philips NV, Amsterdam, The Netherlands) with a 20-channel head coil. Each functional run consisted of 360 T2*-weighted echoplanar images (EPIs). The imaging parameters were as follows: 3 × 3 mm in-plane voxel size, covering the whole brain volume in 4-mm slices; interslice gap = 0 mm; repetition time (TR) = 2000 ms; echo time (TE) = 30 ms; and 76 × 74 matrix. The total functional scan duration was 12 min. In addition to the functional images, we collected a high-resolution T1-weighted anatomical scan for each participant (179 slices, resolution 1 × 1 × 1 mm, TR = 8.2 s, TE = 3.8 ms, 240 × 222 acquisition matrix). The participants were instructed to relax and lie still.

### 2.4. Functional Data Preprocessing Pipeline and Statistical Analysis

The data were processed using the SPM12 (http://www.fil.ion.ucl.ac.uk/spm/, accessed on 6 January 2023) statistical processing package on the MATLAB platform (version 2019b; MathWorks). The preprocessing procedure consisted of realignment of functional images (motion correction), co-registration, segmentation of structural data, normalization into standard stereotactic Montreal Neurological Institute (MNI) space, and spatial smoothing with a Gaussian kernel of 8 mm in full width at half maximum. Statistical parametric maps for fMRI were constructed using a general linear model (GLM) [32]. The GLM model included contrasts to obtain the mean of each stimulus type: stroking the shin with a feather, stroking the shin with the hand, stroking the foot sole with a feather, and stroking the foot sole with the hand for each individual subject. The motion parameters obtained during data preprocessing were included into the first-level model as nonsense covariates. In addition, the data on motion parameters obtained during the realignment steps were compared between different types of stimulation. No significant differences in these parameters between states, including baseline, were found. The individual difference maps were analyzed using within-subjects analysis of variance (RM ANOVA 2 × 2), with two factors for within-group variability: “Body part” (Shin, Sole) and “Type of stroking” (Hand, Feather). To assess correlations of stimulation-related BOLD activity with self-report data, we used a voxel-wise whole-brain approach. We used subjective stimulus evaluation parameters (two parameters (pleasantness and tickling) for each stimulation type and fMRI contrast respectively) to separate multiple regression analyses using all fMRI contrasts. All fMRI group results were thresholded at *p* < 0.05 corrected for multiple comparisons (family-wise error, FWE, correction at cluster level). A correction for multiple correlations was performed. Results with a pFWE < 0.003 at the cluster level were accepted. Anatomical regions of the brain that included activated clusters were identified using the Neuromorphometrics atlas built into the SPM12.

### 2.5. Evaluation of Subjective Perception of Pleasantness and Ticklishness of the Stimuli Presented during fMRI

After completion of the fMRI scans, the subjects were asked to rate all types of strokes experienced for pleasantness and tickling sensation on a 9-point scale, where a value of 5 corresponded to a neutral rating. A total of eight subjective stimulus evaluation parameters (four for tickling and four for pleasantness) were collected from each subject. During debriefing, the subjects were reminded of the type of touch by appropriate stroking. After repeating/reminding the type of stimulation, the subject was asked, “Rate the pleasantness/ticklishness of this stimulus on a scale from 1 to 9, where 5 is a neutral rating. 0—very unpleasant/not at all ticklish, 9—very pleasant/very ticklish”. Statistical analysis of the data was performed using Wilcoxon’s matched pairs test with a significance level of 0.05.

## 3. Results

### 3.1. fMRI ANOVA

**Figure 1:**
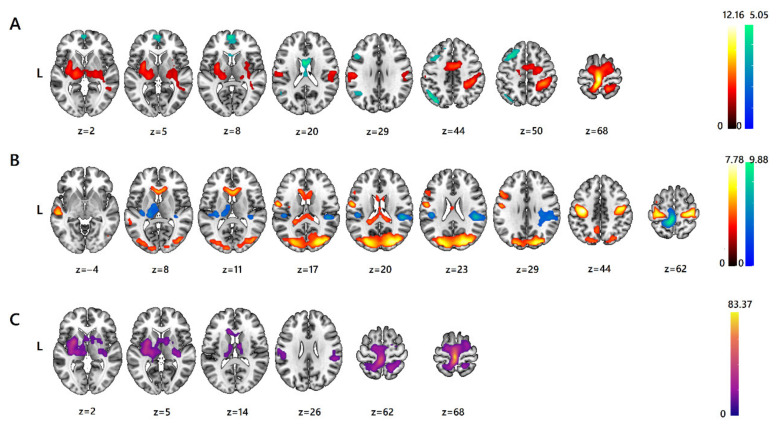
fMRI ANOVA results. Brain activations showing a significant main effect of: (**A**) body part, contrast “Foot sole > Shin” shown in red and “Shin > Foot sole” shown in blue; (**B**) type of stroking, contrast “Peacock feather > Hand” shown in red and “Hand > Peacock feather” shown in blue; and (**C**) interaction of factors “body part × type of stroking”. Maps are thresholded at pFWE < 0.05, (correction at cluster level, cluster forming threshold of *p* < 0.001 uncorrected, min. cluster extent: k = 72 voxels). The color scale represents t-scores. Color bars represent post-hoc t-statistics.

Table 1 and Figure 1 present summaries of the fMRI ANOVA results. A main effect of body part (foot sole versus shin) was the result of an increased BOLD signal for the foot sole stroking in the medial segment of precentral gyri, left precentral gyrus (MI), bilateral putamen and anterior insula, left postcentral gyrus (SI), right thalamus, and pallidum. An increased BOLD response for the shin stroking was observed in the lateral ventricle area and left middle cingulate gyrus, left angular gyrus, left middle frontal gyrus (frontal eye field—FEF), bilateral medial segment of superior frontal gyrus (anterior prefrontal cortex—AntPFC), and left frontal pole (see Figure 1A and Table 1).

A main effect of type of stroking was found with greater BOLD activity for feather stroking compared to hand stroking occurring in the pre- and postcentral gyri bilaterally (premotor and supplementary motor cortex, MI region), superior and middle occipital gyri bilaterally, left middle and superior temporal gyri, right anterior cingulate gyrus and caudate, left middle and inferior frontal gyri, and left lateral ventricle area. Hand stroking evoked greater BOLD activity than feather stroking in the left medial segment of the pre- and postcentral gyri, left postcentral gyrus and precuneus, bilateral parietal operculum, right planum temporale, left central operculum, and left thalamus proper (see Figure 1B and Table 1).

**Figure 2:**
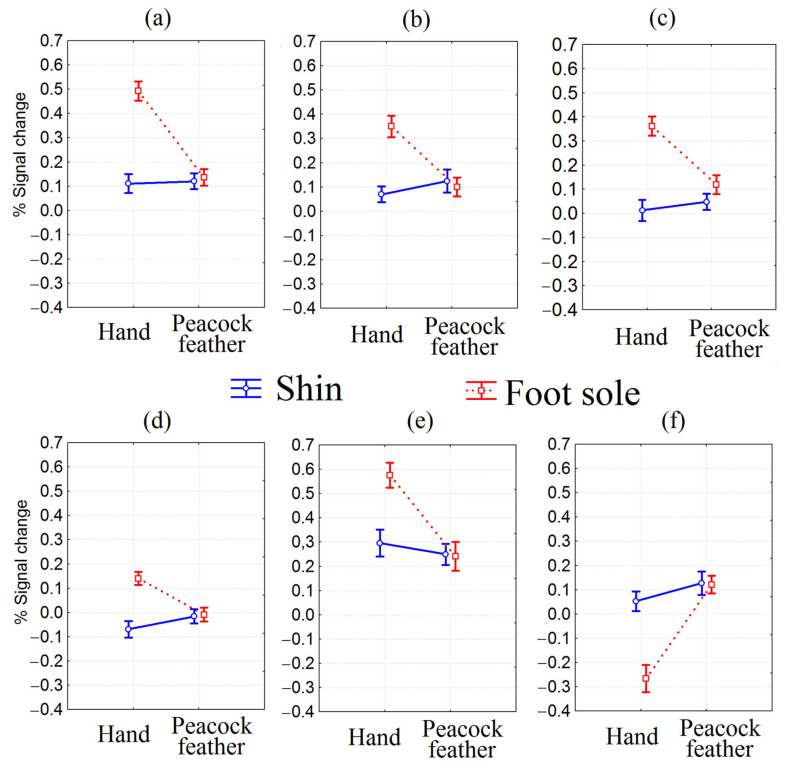
Peak voxels showing a significant interaction between body part and type of stroking (F(1,24) > 24.31, *p* < 0.0001): (**a**) % signal change for cluster bilateral precentral gyrus medial segment and left MI region; (**b**) % signal change for cluster left putamen, pallidum and anterior insula; (**c**) % signal change for cluster right superior parietal lobule; (**d**) % signal change for cluster right transverse temporal gyrus and posterior insula; (**e**) % signal change for cluster right parietal operculum, supramarginal gyrus and planum temporale; (**f**) % signal change for cluster left lateral ventricle and caudate.

Six brain regions showed a significant interaction of body part with type of stroking (see Table 1, Figure 1C and Figure 2). These areas included the bilateral medium segment of precentral gyrus and left precentral gyrus; left putamen, pallidum and anterior insula; right superior parietal lobule; right transverse temporal gyrus and posterior insula; right parietal operculum, supramarginal gyrus, and planum temporale; and left lateral ventricle and caudate. All regions except the last one showed the same tendency of signal change: the percentage signal change was significantly higher for the foot sole compared to the shin only during stimulation by hand (mixed effect F(1,24) > 24.71, *p* < 0.0001; partial eta-squared > 0.51, power 1). The percentage signal change for the left lateral ventricle area and caudate was significantly higher for the shin compared to the foot sole only during stimulation by hand (mixed effect F(1,24) = 24.31, *p* < 0.0001, partial eta-squared 0.50, power 1).

### 3.2. Correlation of BOLD-Signal with Subjective Stimuli Assessment

**Figure 3:**
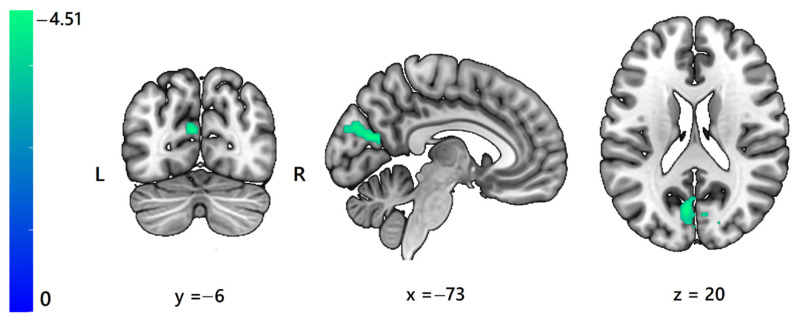
Activation maps for whole-brain voxel-wise multiple regression analysis using the fMRI contrasts and the results of the relevant questionnaires. Brain activation for the contrast “feather foot sole stimulation > baseline” shown as a function of subjective tickling sensation rating in response to the feather sole stroking for all participants. Activated regions (left and right cuneus, left precuneus, left calcarine cortex) are in blue. Maps are thresholded at pFWE < 0.05 (correction at cluster level, cluster forming threshold of *p* < 0.001 uncorrected, min. cluster extent: k = 123 voxels). The color scale represents t-scores.

BOLD signal changes in one cluster showed significant, negative correlations with the subjective tickling sensation of the foot sole region (Figure 3). It included the left and right cuneus, left precuneus, and left calcarine cortex (t(17) = −4.51, p_corr_ = 0.004), with peak activation localization [−6, −73, 20] and cluster size = 123 voxels. Multiple regression of activation maps and pleasantness questionnaire data revealed no significant correlations.

### 3.3. Subjective Perception of Pleasantness and Ticklishness of the Stimuli Presented during fMRI

**Figure 4:**
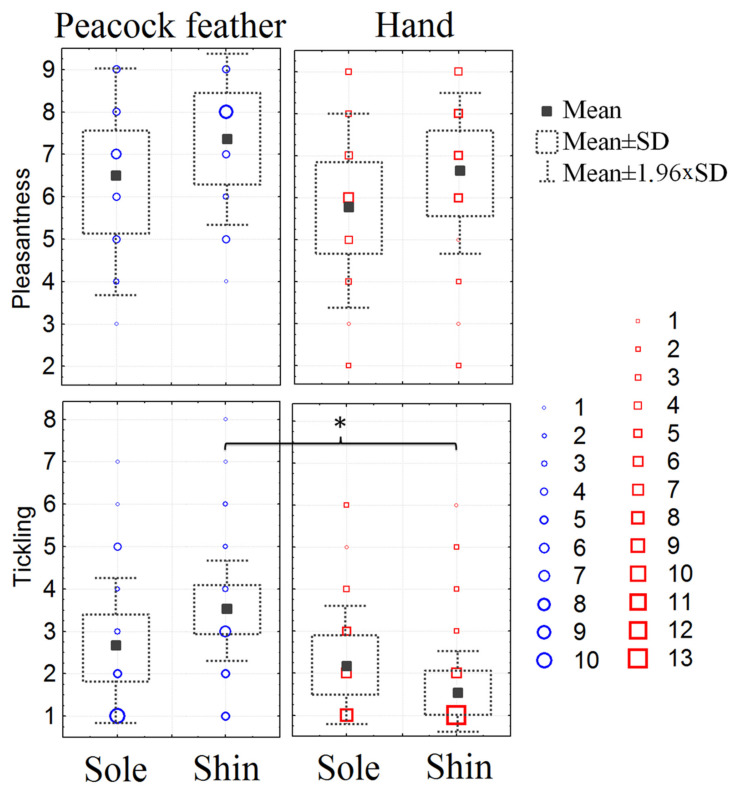
The combination of box plots (mean, SD) and frequency scatterplots (with individuals scores of participants by the scales for “Pleasantness” and “Tickling”) for two type of stimuli (peacock feather and hand) and two area of stimulation (shin and foot sole). Digits 1, 2 … 13—the number of participants with the particular subjective ratings for 26 participants. *—Post hoc Bonferroni’s test *p* < 0.05.

Most subjects rated the feather and hand touches on both the shin and foot sole as pleasant or neutral (the lowest median was equal to 7 (IQR = 2.6) for foot sole hand stroking). There was a tendency to feel the feather touch as more pleasant for both the shin and the foot sole (Figure 4). Most subjects did not find the stimulation ticklish (for all means of stimulation, the mean scores did not reach the mean value (5) on a scale of 1 to 9). None of the subjects suppressed laughter or felt the urge to laugh while being touched. The assessment of stimuli by the scales of tickling and pleasantness showed significant differences only for tickling rates (see Figure 4). The assessment scores by “tickling” scale were significantly higher for the peacock feather than the hand during stimulation of the shin (mixed effect F(1,24) = 5.09, *p* = 0.0335 (partial eta-squared 0.174, power 0.58, post hoc Bonferroni’s *p* = 0.0038). The tickling rates during feather stimulation also differed between the sole and shin and were significantly higher for the shin (post hoc Bonferroni’s *p* = 0.0038, *p* = 0.0089).

## 4. Discussion

Our pilot study aimed to characterize the central mechanisms of processing social and nonsocial stimuli by hairy and glabrous skin of the lower extremities, as well as tickle sensation. We obtained overall more pronounced activation during foot sole stimulation in somatosensory (BA 1) and somatomotor (BA 4) areas more in the left, left anterior insula, and right subcortical nuclei (thalamus proper and pallidum) and bilateral putamen. For the hairy shin area, activation of the left middle cingular gyrus, FEF, frontal pole, bilateral anterior PFC, and left angular gyrus was predominant. The nonsocial stimulus type (peacock feather) activated more the bilateral primary motor cortex (MI), bilateral middle and superior occipital gyri, left middle and superior temporal gyri, right anterior cingulate gyrus and caudate, left middle, inferior (opercular part) frontal gyri, and right lateral ventricle area. Social touch (bare hand) elicited relatively greater activation of the left (contralateral) primary motor cortex (MI), bilateral parietal operculum, right planum temporale, left central operculum, and left thalamus proper. The foot region in terms of differentiating the cortical response appeared more sensitive to discriminating the two types of stimuli. Marked differences in the BOLD response (greater for hand stimulation) were observed in the bilateral precentral gyrus medial segment and left MI region; left putamen, pallidum, and anterior insula; right superior parietal lobule; right transverse temporal gyrus and posterior insula; and right parietal operculum, supramarginal gyrus, and planum temporale. Hand stimulation of the shin caused more pronounced activation in the left caudate nucleus region. The tickling sensation in the foot correlated with the signal level in the cuneal area.

### 4.1. Stimulation Area

Our findings demonstrated that tactile stimulation of the foot sole induced more distributed BOLD activity compared to the shin. Independently of the stimulus type for sole stimulation, we found higher activity of the somatosensory (BA 1) and somatomotor (BA 4) areas more in the left anterior insula and right subcortical nuclei (thalamus proper and pallidum). For the hairy shin area, predominant activation of only cortical areas of the frontal (left middle cingular gyrus, FEF, frontal pole, bilateral anterior PFC) and parietal (left angular gyrus) lobe was shown.

This result seems to be in general agreement with the data available to date regarding the central mechanisms of tactile stimuli processing in the hairy and glabrous skin of the arm. On the one hand, such studies have also obtained preferential activation of the somatosensory cortex during palm stimulation, as well as frontal activation during forearm stimulation [3,33]. Greater activation of the contralateral anterior insula when stroking the glabrous skin of the palm was also obtained in a study by McGlone (2012) [33].

On the other hand, thalamic activation was observed only contralaterally in all known studies, unlike ours [8,9]. This fact raises an issue of laterality that demands further exploration in future studies. Additionally, no studies in this area have to our knowledge shown FEF activation. The FEF area is traditionally associated with eye movements and high-level cognitive processes. However, there is evidence that the human FEF is a remarkably quickly activated multimodal region that belongs to a network of low-level neocortical sensory areas [34]. The more pronounced activation of this region specifically for the shin region may indicate overall less automated, more cognitively expensive perceptual processing, and this result is inconsistent with the hypothesis that pleasant touching of hairy skin, mediated by CT afferents, is processed in the limbic-related cortex and represents an innate non-learned process.

In general, we can say here that the tactile system for the lower extremities has almost the same patterns of representation in the cortex.

### 4.2. Stimulus Type

Peacock feather stimulation (non-social), regardless of the touch area, induced more distributed BOLD activity than bare hand stimulation. We found increased activation in the bilateral primary motor cortex (MI), bilateral middle and superior occipital gyri, left middle and superior temporal gyri, right anterior cingulate gyrus and caudate, left middle, inferior (opercular part) frontal gyri, and right lateral ventricle area. Interestingly, some of these areas, according to other studies, are attributed to the social touch analysis system, the so-called social brain [35]. These areas include one that play a role in analyzing and implementing the spectrum of social interactions: the medial prefrontal cortex, the anterior cingulate cortex, the inferior frontal gyrus, the superior temporal sulcus, the amygdala, and the anterior insula [36]. The STG is an area known to track social perception [37,38]. No predominant activation of these brain areas was observed for hand touch. The areas that showed increased activation for hand (social) touch compared to feather touch were the left (contralateral) primary motor cortex (MI), bilateral parietal operculum, right planum temporale, left central operculum, and left thalamus proper. Our results are in contrast to the results of other studies in which skin-to-skin touch was used. For example, in Strauss et al. (2019), a comparison of brain activations for interpersonal and impersonal touch in the healthy group revealed greater activation in the right precentral gyrus extending to right postcentral gyrus [22], nor was greater activation in the insula region observed as in [23]. Moreover, in our study, the anterior cingulate cortex, which is strongly activated by skin-to-skin touch in the forearm region [12], was more activated by the impersonal peacock feather touch. We want to point out possible evolutionary reasons for the results obtained. The skin of the shin and foot sole should be evolutionarily less adapted to the recognition of social affective touches, while the analysis of the knismesis touch and the timely elimination of its cause (snakes, dangerous insects, etc.) could play more important roles in the survival of the specimen. Obtained activation of the areas associated with the “social brain” to non-social stimulation in the lower limb may, in our opinion, provide some food for thought concerning the neuronal basis and development of the social tactile perception system in the brain.

Given that both types of stimuli were CT-directed, it can also be assumed that the C-tactile afferents from the foot sole come to the cortex not as specialized as those for the arm in terms of the “sociality” of the stimulus.

### 4.3. Interaction of Factors

We identified brain regions that are specifically activated by hand stroking in the shin area and in the foot sole area. In terms of this cortical activation, the foot sole region was much more sensitive to differentiating between the social and nonsocial stimuli that we used. Hand stroking of the foot sole region significantly increased the BOLD signal in the following areas: (a) bilateral precentral gyrus medial segment and left MI region; (b) left putamen, pallidum and anterior insula; (c) right superior parietal lobule; (d) right transverse temporal gyrus and posterior insula; and (e) right parietal operculum, supramarginal gyrus, and planum temporale (Figure 2a–e) compared to all other types of stroking. Moreover, only in the (f) left lateral ventricle area and caudate was the activation during stimulation by the hand of the foot sole less than during stimulation by the hand of the shin (Figure 2f). In all the above six brain regions, stimulation with a peacock feather elicited almost the same BOLD-signal when stroking both the foot sole and shin regions.

If one considers that hand strokes are more CT affine than feather strokes and given the inclusion of the posterior insula in the effect observed in the case of the foot sole, this result is intriguing. These findings differed from the well-known data that CT neurons had been found mostly in the hairy skin in humans and, according to microneurography, were not found in glabrous skin of the arm [1,39]. However, while the palm and sole skin has similar anatomical structure, in humans, unlike in other animals, the upper and lower limbs perform fundamentally different functions. Similar to some mammals, humans use their feet to enable locomotion. In this regard, we could hypothesize that, similar to rats, in which CT neurons were found in the glabrous skin of the hind paw [40], humans, although rudimentary, may have CT afferents or their functional homologue in their feet soles rather than in their palms [41]. Recent work by Roger Watkins et al. showed a sparse population of C-tactile afferents in glabrous skin in humans, while it is currently unknown whether it is able to contribute to affective perception of a gentle touch [39].

Thus, it is possible that, due to the specialization of the upper limbs, the migration of CT afferentation from the glabrous skin of the palm to the forearm occurred during evolution, while this process did not occur in the leg region. Thus, CT afferents may evolutionally originate from the glabrous skin of the limbs.

### 4.4. Subjective Perception of Pleasantness and Ticklishness of the Stimuli

We found no significant differences in subjective perception of pleasantness between stimuli and areas of stimulation. The same lack of difference in pleasantness ratings between hairy and glabrous skin has been obtained for the upper limbs [33].The subjective tickling sensitivity score for stroking with the feather was higher for the shin than for the foot. It was also significantly higher for the feather touch than for the hand touch but only on the shin. Studies conducted on the upper extremities have shown that touching the hairy skin of the forearm has greater affective value than touching the glabrous skin of the palm [4,33,42,43]. Another study found that subjects used more sensory descriptors when evaluating palm touch, while they used more emotional descriptors for hand touch [44]. In our study, we obtained the opposite result for the hairy and glabrous parts of the lower extremities. This fact, as well as discreteness exclusively for the ticklishness score and not for the pleasantness score, may again be explained by the evolutionary absence of the social component of perception in this area.

Only the subjective assessment of tickling in the foot sole region when stroking with the peacock feather had a significant correlation with activation in the brain (bilateral cuneus, left precuneus, and calcarine cortex). We see some limitations of our study in terms of subjective evaluation of the stimuli, which we point out later. Nevertheless, we find this result quite interesting given that the precuneus as part of default mode network is involved in integrating cognitive and emotional processing [45], representing pleasant states [46], regulating and processing emotions [47], and representing emotional concepts by instantiating simulations and sending predictions that alter processing through the entire cortical sheet, terminating in the primary sensory and motor regions [48]. In addition, functions associated with the precuneus include attention monitoring and response inhibition, motivation-independent neural process [49], and somatosensory processing. There is also evidence that brain activations in the cuneus region have been associated with fear [50], threats [51], and proneness to anxiety reactions [52,53]. Given these data, a possible explanation for the resulting negative association between subjective evaluation of tickling (not itching) and cuneal area activation may reflect complex emotional processing from stimulus safety or anxiety to suppressing a response to it.

Thus, considering the results obtained, we can say that the C-tactile system of the lower limb has similar patterns of representation in the cortex. At the same time, afferents from the leg come to the cortex not as specialized as those for the hand for social and nonsocial stimuli. Nevertheless, there are areas in the cortex that respond specifically to hand and feather touch in the foot sole region. These areas include the posterior insula, which is considered one of the cortical ends of CT afferents. No such brain areas have been identified for the shin. We also obtained new data on the neurobiological basis of subjective tickle perception in the soleus region and identified the role of the precuneus region in this outcome.

Our results make some contributions to understanding the physiology of the perception of social and non-social tactile stimuli and the CT system, including its evolution. We have also established an area putatively associated with tickle perception in the foot sole area. Our results also have practical implications. The findings on the perception of stimuli by the foot sole could be used for sensory enrichment of patients with a decreased level of consciousness, as well as with sensory loss in the upper limb region. Further research will be aimed at refining the findings.

## 5. Limitations

The main limitation of the study is the relatively small sample size. However, we made an effort to render the demographic characteristics of the subjects homogeneous, and multiple comparison adjustments were made during the statistical analysis. In addition, for technical reasons, the subjective evaluation of the stimuli was conducted at the end of the study, rather than as responses after each block of stimulation, which would have been more relevant. We also administered a tickling questionnaire, although it might have been more relevant to also question the subjects about the sensation of itching, given the knismesis type of peacock feather stimulation. A closed-ended question about the degree of tickling could, first, prevent the subject from more accurately assessing the nature of the sensation being tested; and second, automatically direct the subject’s attention to seeking some positive emotion (associated, for example, with laughing, which is routinely found in connection with tickling) from the stimulus. In addition, given the large difference in pressure force between feather stimulation and hand stimulation, the subjective perception of pressure force should also be questioned. Our future work in this area will focus on increasing the statistical power by increasing the sample size and on increasing the sensitivity of subjective evaluation of stimuli.

## 6. Conclusions

Touching the foot sole resulted in widespread activation in the brain that was clearly differentiated from affective touching of the hairy shin skin. Given the areas involved, this differentiation generally reflected likely less automatic processing of stimuli by the skin of the shin. Stimulation by nonsocial stimuli in the lower extremities paradoxically resulted in greater activation of brain regions intrinsic to the perception and processing of social stimuli in general. There are regions in the cerebral cortex that responded specifically to hand and feather touches in the foot sole region. These areas included the posterior insula, which is thought to be one of the cortical ends of the CT afferents. No such brain areas have been identified for the shin. For subjective evaluation of stimuli on the lower extremities, there was no differentiation for social and nonsocial CT-directed stimuli in terms of pleasantness. There was a negative correlation of subjective perception of ticklishness from a feather in the foot sole area with the strength of activation in the cuneal area.

Our results have implications for understanding the basics of analyzing CT-targeted stimuli in the lower limbs and may further serve to understand the evolutionary development of this human body system. From a practical point of view, our results may be of interest in the development of the sensory stimulation of patients with long-term disorders of consciousness, as well as with loss of sensation or inaccessibility of the upper limbs for contact.

## Figures and Tables

**Table 1:** Brain activations (ANOVA results) showing a significant, main effect of body part and type of stroking (F(1,75) > 20.24, corrected *p* < 0.05). AntPFC, anterior prefrontal cortex, FEF—frontal eye field; MI, primary motor cortex, PO, parietal operculum, PT, planum temporale, SI, primary somatosensory cortex.

Brain Region (and Functional Localization)	L/R	Cluster Size (Voxels)	Local Maxima (MNI)			BA	*p*-Value (Cluster-Level, FWE *p* < 0.05)	F-Value	Direction (Post-Hoc)
			x	y	z				
*Main effect: Foot sole* vs. *Shin*									
Precentral g. medial segment Left precentral g. (MI)	L, R	1986	−3	−28	68	4	0.000	147.96	Sole > Shin
Putamen Anterior insula	L	315	−30	−1	5	–	0.000	33.87	Sole > Shin
Postcentral g. (SI)	L	148	−51	−19	29	1	0.003	26.20	Sole > Shin
Lateral ventricle Left middle cingulate g.	L, R	142	−3	5	20	–	0.003	25.54	Shin > Sole
Angular g.	L	153	−48	−64	44	39	0.002	22.85	Shin > Sole
Thalamus proper Putamen Pallidum	R	143	24	−16	2	–	0.003	22.27	Sole > Shin
Middle frontal g. (FEF)	L	158	−33	17	50	8	0.002	20.99	Shin > Sole
Superior frontal g. medial segment (AntPFC) Left frontal pole	L, R	75	−3	59	8	10	0.038	20.24	Shin > Sole
*Main effect: Feather* vs. *Hand*									
Postcentral g. medial segment Postcentral g. Precuneus Precentral g. medial segment (MI)	L	668	−6	−40	65	4	0.000	97.62	Hand > Feather
Precentral g. (MI) Postcentral g.	L	615	−36	−19	44	4	0.000	60.58	Feather >Hand
Parietal operculum (PO) Planum temporale (PT)	R	313	48	−31	23	40	0.000	53.28	Hand > Feather
Precentral g. Postcentral g.	R	450	36	−22	62	6	0.000	51.94	Feather >Hand
Superior occipital g. Middle occipital g.	R, L	1455	27	−82	20	19	0.000	48.98	Feather >Hand
Parietal operculum Central operculum	L	106	−45	−25	23	–	0.011	35.40	Hand > Feather
Middle temporal g. Superior temporal g.	L	81	−54	−25	−4	22	0.030	33.55	Feather >Hand
Anterior cingulate g. Caudate	R	157	6	23	11	–	0.002	30.69	Feather >Hand
Thalamus proper	L	142	−18	−25	8	–	0.003	26.09	Hand > Feather
Middle frontal g. Inferior frontal g. opercular part	L	86	−54	20	29	44	0.025	22.41	Feather >Hand
Lateral ventricle	L	85	−21	−40	17	–	0.026	20.79	Feather >Hand
*Interaction of factors (“Body Part”* × “*Type of stroking”)*									
Precentral g. medial segment Left precentral g. (MI)	L, R	1673	−3	−28	68	4	0.000	83.37	–
Putamen Pallidum Anterior insula	L	718	−27	2	2	–	0.000	41.99	–
Superior parietal lobule	R	245	18	−55	62	7	0.000	28.19	–
Transverse temporal g. Posterior insula	R	83	36	−25	5	–	0.028	24.15	–
Parietal operculum (PO) Supramarginal g. Planum temporale (PT)	R	147	54	−34	26	40	0.003	23.72	–
Lateral ventricle Caudate	L	72	−12	23	14	–	0.043	23.58	–

L—left, R—right.

## Data Availability

The raw data supporting the conclusions of this article will be made available by the authors without undue reservations.
